# “Whatever happens, happens” challenges of end-of-life communication from the perspective of older adults and family caregivers: a Qualitative study

**DOI:** 10.1186/s12904-019-0493-7

**Published:** 2019-12-12

**Authors:** Jennifer Im, Susanna Mak, Ross Upshur, Leah Steinberg, Kerry Kuluski

**Affiliations:** 1 Institute of Health Policy, Management, and Evaluation, University of Toronto, Canada155 College Street – Suite 425, Toronto, Ontario M5T 1P8 Canada; 2 Division of Cardiology, Mount Sinai Hospital, Sinai Health System, Toronto, Ontario Canada; 3 Institute of Medical Sciences, University of Toronto, Toronto, Ontario Canada; 4 Lunenfeld-Tanenbaum Research Institute, Sinai Health System, Toronto, Ontario Canada; 5 Dalla Lana School of Public Health, University of Toronto, Toronto, Ontario Canada; 6 Department of Family & Community Medicine, University of Toronto, Toronto, Ontario Canada; 7 Temmy Latner Centre for Palliative Care, Mount Sinai Hospital, Sinai Health System, Toronto, Ontario Canada; 8 Institute for Better Health, Trillium Health Partners, Mississauga, Ontario Canada

**Keywords:** End-of-life communication, Palliative care, Older adults, Caregivers, Serious illness, Heart failure, Qualitative research

## Abstract

**Background:**

Effective communication is integral to the delivery of goal-concordant care for older adults and their family caregivers, and yet, it is uncommon in people with serious illness. This study explores the challenges of integrating end-of-life communication into heart failure management from the perspectives of older adults and family caregivers.

**Methods:**

In a qualitative study of older adults with heart failure and their family caregivers, fourteen semi-structured interviews were conducted with 19 participants in Ontario, Canada. The interviews were transcribed verbatim and thematic analysis was applied to analyze the data.

**Results:**

Four themes were identified in the context of participants’ understanding of illness: 1) trivializing illness-related challenges, 2) positivity in late life, 3) discomfort in having end-of-life conversations, and 4) reluctant to engage despite need. These challenges often intertwine with one another. Most participants had not engaged in end-of-life discussions with their clinicians or family members.

**Conclusion:**

The findings provide insights that can inform approaches to integrate end-of-life communication for older adults with serious illness and caregivers. The identified challenges highlight a need for end-of-life communication to occur earlier in illness to be able to support individuals throughout the period of decline. In addition, end-of-life communication should be introduced iteratively for those who may not be ready to engage. Alternative approaches to communication are needed to elicit the challenges that patients and caregivers experience throughout the progression of illness to improve care for people nearing the end of life.

## Background

Effective communication is integral to the delivery of goal-concordant care for older adults as they progress throughout their illness to the end of life. Having goals-of-care discussions is one way to achieve goal-concordant care; this is part of the end-of-life communication process, which aims to create a shared understanding of an individual’s goals and values among patients, family members and clinicians [1]. However, evidence suggests that these important conversations occur infrequently for patients and caregivers [2]. For older adults with heart failure and other chronic conditions, the nature of illness underscores the need for earlier communication given the intermittent decline patients and families experience.

Chronic heart failure is a progressive illness characterized by an uncertain clinical trajectory, and high risk of morbidity and mortality that affects over 8% of older adults over the age of 75, globally [3]. Many patients experience interruptions to their quality of life given the high symptom burden such as edema, and fatigue [4–7]. In older adults, understanding and management of illness are also challenged by the presence of frailty, multimorbidity, and polypharmacy. In the United States, patients with heart failure have, on average, 5 chronic conditions at a given time [8, 9], are prescribed up to an average of 11 medications [10], and up to 76% of older adults have been reported to be frail [11]. Therefore, the complexity of illness poses challenges for clinicians, patients and family caregivers to engage in end-of-life communication all the while highlighting the need for earlier end-of-life communication in older adults with heart failure.

There are additional barriers of end-of-life communication in healthcare. At the system level, a lack of integration in clinical care and a lack of interoperability in electronic health records presents challenges for clinicians to collaborate in patient care [12, 13]. At the same time, clinicians lack clarity as to which provider in the patient’s care should engage in end-of-life communication [14]. Despite recommendations from multiple medical associations in cardiology to integrate end-of-life communication early in the illness trajectory [15–18], cardiologists often assign the responsibility of end-of-life discussions to other disciplines such as palliative medicine and primary care [14], in part because clinicians feel underprepared to initiate end-of-life discussions [19, 20]. Above all, clinicians have reported patient- and family- related factors to be the most important barriers of end-of-life communication [21]. In a survey of clinicians in cardiology, You and colleagues reported difficulty with accepting prognosis and lack of understanding of end-of-life treatments among patients and family members as the most important barriers of goals-of-care discussions. Other studies have similarly reported lack of understanding of illness and prognosis, misaligned expectations of outcomes, and disagreement about goals of care among patients and caregivers as barriers of end-of-life communication [22–25].

Nonetheless, the perceptions of engaging in earlier end-of-life communication from the perspective of older adults with serious illness and their family caregivers remain poorly understood. If patient and caregiver- related barriers are the most important barriers, then, their perspectives can yield valuable insights to inform clinical approaches to improve the integration of end-of-life communication in serious illness. To address this gap, this study explores the challenges of end-of-life communication from the perspectives of older adults with heart failure and their family caregivers. This is a sub-study of a larger study that explored patient and caregiver perceptions and understanding of illness, goals of care, and engaging in end-of-life discussions in advanced heart failure.

## Methods

### Design, setting, and participants

This study was guided by interpretive description methodology, which acknowledges the constructed and context-dependent nature in which health-related experiences form [26, 27]. Using a purposeful sampling technique, older adults and their family caregivers were recruited from a specialized outpatient heart failure clinic in a medium-sized urban teaching hospital in Ontario, Canada (see Table 1 for inclusion criteria). An interdisciplinary team of cardiologists, a nurse practitioner, and a pharmacist, all of whom have specialized training in heart failure, care for patients in the clinic. Over the course of recruitment, 36 patient candidates were identified. Active recruitment concluded after 22 patients were contacted to participate in the study. Of the 22 study candidates that were contacted for recruitment, two patients declined to be approached by the researcher and one candidate could not be approached due to cognitive decline. Recruitment of study participants came to an end when thematic saturation was reached which was determined based on no new data on relevant phenomena of interest being generated. No participants withdrew from the study.

**Table 1:** Inclusion criteria for participant recruitment

• Patients aged 65 years and older with advanced heart failure (NYHA Class III/IV) or aged 80 years and older under care in the heart failure clinic	
• Not waiting for a transplant	
• Not under care by a specialist palliative care provider	
• Have adequate stamina to complete an hour-long interview	
• Able to provide informed consent	
• English speaking	

### Data collection

In-person, semi-structured interviews were conducted with older adults and their family caregivers as individual or dyad interviews. Interview guides were developed by the lead author (JI) with input from the multidisciplinary research team (i.e. clinicians and researchers in cardiology, palliative care, family medicine and social work). The interview guides included open-ended questions about participants’ understanding of illness, their goals of care, and prior engagement in end-of-life discussions. Probes were used to generate discussion on specific aspects of end-of-life communication such as hypothetical scenarios and concerns about the future (see Additional file 1 for interview questions). Adaptations were made to the interview guides as data collection and analysis progressed to refine questions and exhaust emerging concepts.

Interviews were conducted by a trained qualitative researcher (JI) who was mentored by the senior author (KK), an experienced qualitative researcher who leads a program of research on the experience of vulnerable populations and their caregivers. Participants were introduced to the study and provided an opportunity to ask questions. The data collection process began by collecting participant characteristics followed by semi-structured interviews. The interviews were audio-recorded and transcribed verbatim by a medical transcriptionist and participants were reminded that their participation was voluntary. To ensure data accuracy and confidentiality, the transcripts were checked against the original recordings and identifiable information was redacted. The majority of the participant interviews were conducted at the clinic in a private room and the remainder of the interviews were conducted in participants’ homes based on their preference. For patients and caregivers who were recruited as dyads, the decision to be interviewed individually or together was left to the participants given the sensitivity of the topic under inquiry. No prior relationship between the interviewer and any participants existed. The interviews ranged from 25 to 90 min, although most interviews were completed in 45–60 min. All interviews were completed in English in one session. The data collection period lasted from August 2017 to January 2018.

### Data analysis

The analytical process began with reviewing the transcripts at a high-level alongside data collection. To become familiar with the data, transcripts were reviewed and data were coded using a qualitative data management software program, NVivo11 (QSR International). Upon becoming familiar with the data, the first author reviewed the transcripts in depth by applying the specific research question under inquiry. Thematic analysis was used to code the data in order to identify patterns of related constructs to generate codes and themes [28]. Descriptions and interpretations of different excerpts were juxtaposed throughout the transcripts to answer the research question. While reviewing each transcript, relevant data that related to barriers of end-of-life communication were extracted and coded.

To ensure rigour, the process of developing themes and interpreting the findings consisted of reviewing the transcripts multiple times. A qualitative memo was created to document the coded excerpts and their accompanying interpretation, which was reviewed by the senior author (KK). Regular meetings were held with the team as well as knowledge users (e.g. clinicians in cardiology and palliative care) to discuss findings as analysis progressed and as themes became identified. Engaging with the research team and the knowledge users helped to increase the trustworthiness and credibility of the findings as multiple perspectives from various disciplines were accounted for.

### Ethics

Prior to data collection, the ethics application was approved from the Research Ethics Boards at Sinai Health System (July 12, 2017). All study participants provided written informed consent to participate in the study, which included consent for audio recording and transcription of the interview, and for the findings to be used in the dissemination of knowledge upon removing identifiable data.

## Results

Fourteen interviews were conducted with 19 participants (7 solo interviews and 5 dyad interviews; 12 patients and 7 family caregivers). The mean age of patients was 82.5 years (SD = 6.4); the majority were male (58%), reported a mean of 5 chronic conditions (SD = 2.2), lived with their spouse (58%), and were predominantly Caucasian (92%) (see Tables 2 and 3). The mean age of caregivers was 67 years (SD = 13.7); the majority were female (71%) and most were spouses of patient participants (71%) (see Table 4).

**Table 2:** Patient Characteristics (*n* = 12)

Characteristics	Patients (%)
Gender	
Male	7 (58%)
Age	
Mean	82.5 (±6.4)
65–74	3 (25%)
75–84	2 (17%)
≥ 85	7 (58%)
Marital Status	
Married	7 (58%)
Other (e.g. widowed, divorced)	5 (42%)
Ethnicity	
Caucasian	11 (92%)
Other	1 (8%)
Living Alone	
No (e.g. with spouse or family)	8 (67%)
Type of Home	
Single/Family home	5 (42%)
Apartment	7 (58%)

**Table 3:** Patient-reported chronic conditions

Chronic conditions	Patients (%)
Mean	5 (±2.2)
Hypertension	9 (75%)
Hyperlipidemia	6 (50%)
Asthma	1 (1%)
Diabetes	4 (33%)
Stroke	1 (1%)
COPD*	1 (1%)
Renal disease	5 (42%)
Liver disease	0 (0%)
Cancer	1 (1%)
Anxiety/depression	1 (1%)
Dementia/Alzheimer’s	0 (0%)
Arthritis	8 (67%)
Osteoporosis	5 (42%)
Mental/cognitive illness	3 (25%)
Other	4 (33%)

*COPD: chronic obstructive pulmonary disease

**Table 4:** Family Caregiver Characteristics (*n* = 7)

Characteristics	Caregivers (%)
Gender	
Female	5 (71%)
Age	
Mean	67.0 (±13.7)
< 65	3 (43%)
65–74	2 (29%)
75–84	0 (0%)
≥ 85	2 (29%)
Relationship to Patients	
Spouse	5 (71%)
Child	2 (28%)
Living with Patient	
Yes	7 (100%)

Four main challenges of engaging in end-of-life discussions were identified: trivializing illness, positivity in late life, discomfort in having end-of-life conversations, and reluctant to engage despite need (additional quotes can be found in Table 5). Although four challenges are discussed independently, these challenges are intertwined with one another. The challenges are identified in the context of participants’ understanding of illness, which is reported in a separate article [29]. Briefly, participants had detailed knowledge of heart failure management and its self-care behaviors, and yet, participants appeared limited in their understanding of the consequences of illness and its progressive nature. Participants did not connect declines in health as part of the progressive illness trajectory; rather declines in health were perceived as temporary health states and hospitalizations were thought to be a routine part of illness management. Despite challenges they faced, participants adapted to the challenges of heart failure, which appeared to influence their perception of overall health. Most participants had not engaged in prior end-of-life discussions.

**Table 5:** Challenges of Engaging in End-of-Life Communication

Themes	Participant Information	Quotations
Trivializing Illness	PT07: 90 years old, 5 chronic conditions	“...I can go into the kitchen, and I stand there…And I’m exhausted. And I have to lean on the counter until I’ve got everything together…But it knocks me out... But once I sit down, I’m fine again. I regain everything. So I’m all right here…and I think, oh, I’ve just got to get upstairs and get a cup of tea or something, or I want a glass of water, and it’s like pulling teeth…But up to now, I’ve been managing.”
Positivity in Late Life	PT06: 76 years old, 6 chronic conditions	“When you are 77 years old, it’s very important for you to have positive things. So you gotta think about positive things, don’t think about the negative.”
	PT11: 74 years old, 5 chronic conditions	“I realize that I haven’t got much longer. And I think that’s about the best way for me to look at it. Like I know it’s not going to…I’m not going to be here when I’m 80. So that’s it. But until there, I’m going to live the way I am now and not let it get me down”
	PT12: 87 years old, 6 chronic conditions	“It’s not part of my thoughts – dying. I mean I don’t want to think about it. I know it’s inevitable but I don’t want to sit and dwell upon it. Whatever happens will happen. And that’s the way I feel”
Discomfort in Having End-of-Life Conversations	CG10: 55 years old, caregiver of patient 10, 88 years old, 4 chronic conditions	“For me, having that kind of conversation with, except for her GP who she sees pretty regularly for one thing or another, they don’t know us well enough on that level to have that kind of conversation about end of life… Somebody that I see every 3 to 6 months or less is not a person that I think I want to talk about my mom’s end of life with. Maybe her GP because she’s had her GP for a long time…or for quite a while, and she sees him quite a bit.”
Reluctant to Engage Despite Need	CG07: 54 years old, male, caregiver of PT07, 90 years old, 5 chronic conditions	PT07: It’s like [Daughter] said, “Look at the sunset, mom.” I can’t see the sunset.CG07: Right. So if it gets to the point where the very basics are not…you can’t enjoy the basics, that concerns me... it’s simply that she’s not in any severe pain but also managing…finding food, you know, that you like to eat… Foods that you can eat, that you like to eat…picking activities that you can do…it’s the quality of life as much as it wouldn’t appear to be so, is above your pain management, above your general health management, for me takes as much time.
	CG10: 55 years old, female, caregiver of patient 10, 88 years old, 4 chronic conditions	“I do want and expect the healthcare professional to give us a reality check…Like if they say, you know, we could do this heart procedure but really it’s only going to improve it 10% and that’s only going to be very short term, maybe for another 3 months and then you’re going to be back where you were… Because I do see that it’s possible just to continue to do things and do things to keep things going. But if it doesn’t improve the quality of life in any meaningful way…I do expect them to provide that reality check. Because I can’t be impersonal about that. My goal is to keep her going as long as possible with some quality of life in any meaningful way…If it’s only going to be for a small improvement for a short period of time then I need somebody to say that. Because I’m not going to have that objectivity.”

### Challenges of end-of-life communication

#### Theme 1: trivializing illness – “Everything is…I’m managing fine”

Participants described the manifestations of heart failure as interrupting their day-to-day living commonly due to symptoms including fatigue and difficulty walking. Symptoms of illness impeded patients’ ability to perform day-to-day tasks such as cooking and shopping. At the same time, participants described the ways in which the recommended self-care behaviours of heart failure (e.g. dietary restrictions and taking medications) interfered with aspects of life they once enjoyed such as eating their favourite foods. However, even as patients described a loss of independence and certain pleasures in life, they emphasized their ability to continue managing under their circumstances. For example, a 90-year old patient with advanced heart failure, and other chronic conditions described her difficulty with walking up the stairs, which further affected her ability to perform other tasks such as preparing meals for herself. Throughout the interview, the participant reiterated her ability to manage “fine” (PT07). Hence, it appears that despite the difficulties experienced participants have a tendency to maintain their circumstances. As one caregiver put it:

CG03: it’s not enough to say to a patient, “How do you feel?” It’s useless… if his family physician were to phone him or email him and say, “How are you doing?” he’d say, “Fine.” He just wouldn't be around, that’s all. (71y/o, female, caregiver to patient 3, 89 y/o, 7 chronic conditions)

Theme 2: positivity in late life: “don’t think about the negative”

During discussions about the end of life being a near future prospect, participants stressed the importance of maintaining a positive attitude through the period of progression into late life. When asked about their perceptions of engaging in end-of-life discussions, patients thought about the end of life as inevitable but felt they lacked control over related events. For this reason, patients often did not see a point in having end-of-life discussions. Often, patients would say, “whatever happens, happens,” to convey a sense of lacking control as well as a sense of ease with the prospect of death in the near future. Conversations on end-of-life care were uncommon among patients with their family members or healthcare providers. While most patients perceived a lack of need to discuss end-of-life care, a few caregivers acknowledged the importance of discussing end-of-life preferences “because then if suddenly then something happened, and you think, oh, now what am I going to do?” (CG11). Nonetheless, such moments of realism seemed to get pushed aside among caregivers.

PT02: Whatever happens, happens. No good worrying about it, it’s not gonna change too much…you basically forget about it…I figure I do all I can now to keep myself healthy. I eat regular and don’t drink too much and get proper sleep. (85y/o, 2 chronic conditions)

#### Theme 3: discomfort in having end-of-life conversations

For many participants, engaging in end-of-life discussions with family members was thought to be a difficult process. Patients and caregivers felt that end-of-life discussions were uncomfortable to raise with family members, particularly with adult children, as such conversations were thought to bring upon feelings of panic, fear, and grief to their children. Patients also shared that end-of-life care discussions would be met with resistance and unwelcomed by their children such that children would say, “if we mentioned anything like that, our daughter would say, ‘What’s happening...why are you talking about it now?’” (CG12). Consequently, patients and caregivers did not opt to discuss end-of-life care with their family members. Among caregivers, they appeared to be unsure of when or how to engage in end-of-life discussions with their loved ones such that they appeared to be waiting for the opportunity to present itself or had expectations of their clinicians to discuss the end of life, when needed.

PT04: It’s not something that I’m comfortable talking to them about, or are they comfortable talking with me about it. So it’s going to be an obligation they’ll have to deal with, like it or not…It’s an issue which will cause them grief. And I don't want to put grief on them if I can avoid it. So by ignoring it or not dealing with it, you know, we just go through life from day-to-day. (80 y/o, 9 chronic conditions)

Participants had mixed feelings about having end-of-life discussions with clinicians. Some patients and caregivers felt that it was important for their clinicians to know about their values and preferences for care as they approach the end of life. A couple of participants, however, felt that discussions about the end of life were not to be had with their clinicians as a long-standing relationship did not exist with their specialist clinicians and that end-of-life discussions fell outside of the scope of their specialist’s domain of care. Among patients with caregiver participants, all but one dyad had prior end-of-life discussions with family members or clinicians.

#### Theme 4: reluctant to engage despite need

Relatedly, participants appeared initially hesitant to engage in end-of-life discussions. However, caregivers in particular, shared concerns that related to the health of their loved ones. Specifically, caregivers discussed the struggles of managing the increasing needs and supports that accompanied the decline in health and independence. This included being available if and when falls occurred, ensuring that basic needs around day-to-day living and managing the multiple illnesses were met. Furthermore, caregivers were grappling with the distress of their own desire to do right by their loved ones. They wanted to provide their loved ones a life worth living beyond meeting the general basic and medical needs. Nevertheless, caregivers discussed their concerns of what the future would entail as their loved ones decline further and wrestled with their thoughts about how they would manage the challenges that ensue.

CG03: Oh, [sigh] God help me! I have no idea. I do know, and this is a real dilemma for me, is that [Husband] wants…he wants to die at home. And I don't think that I really understood what that meant until recently… I know that if I were to say I can’t do this anymore, it would kill him. It would absolutely kill him. So I’m in a total quandary about this… I don't think he quite gets what it feels like. And it would be a burden to him to communicate that to him. As I say, I mean he can hyperventilate if I’m anxious. So I just don’t push it. Like for what would I be doing? What reason? It’s not a discussion that we can have as equals. And of course that’s part of the end of life too, is it’s no longer the same partnership. (71y/o, female, caregiver to patient 3, 89 y/o, 7 chronic conditions)

## Discussion

In this qualitative study of older adults with heart failure and their family caregivers four challenges of end-of-life discussions were identified: trivializing illness-related challenges, positivity in late life, discomfort in having end-of-life conversations, and reluctant to engage despite need. Uniquely, these challenges were identified within the context of participants’ understanding of illness, which is critical, as it sets the foundation for individuals to perceive a need to engage in end-of-life discussions. Our findings corroborate the work of previous research on the experiences and needs of people with serious illness at the end of life. In a qualitative study of older adults with heart failure, Klindtworth and colleagues reported that participants downplayed their symptoms and the challenges of illness as a process of adapting to living with a serious illness [30]. In a longitudinal qualitative study of 828 interviews with patients with serious illness and their caregivers, Kendall and colleagues also found that participants were reluctant to discuss the end of life, preferred to remain positive, and adapted to new norms as they progressed in illness and faced disabling symptoms [31]. These intertwining challenges point to the need for alternative approaches for communicating with older adults and caregivers. The struggles that older adults and caregivers regularly face in their home environments can be indicative of declines in health and one’s ability to care for themselves. Our study found that caregivers appear to be in distress over managing the challenges of decline, and yet, may be initially reluctant to engage in end-of-life discussions. This underscores the need for more effective communication to elicit the struggles that remain unknown to clinicians during a period of decline. Effective communication may enable clinicians to coordinate services that older adults and families’ can benefit from such as home care, social care services and palliative care. This is important in order to improve support for caregivers of people with advanced heart failure, which has recently been identified as a research priority [32].

Regardless of one’s discipline, providing clinicians with training on effective communication in serious illness is one way to integrate end-of-life discussions earlier in the illness trajectory. Communication is a fundamental competency in medicine, and yet, clinicians repeatedly report feeling underprepared to initiate or have end-of-life discussions [33–35]. In the United States, changes to the undergraduate curricula have led all medical education programs to include provision of at least some material on death and dying. However, less than a quarter of programs offer separate courses on end-of-life care [36]. With an increasing aging population, it ought to be necessary for medical education programs to include rotations in palliative care training for medical students to gain exposure and clinical opportunities to interact with patients and families in real-world end-of-life situations [34]. In fact, desire for such opportunities has been cited in previous research [34, 37]. In addition, interventions to improve communication for both clinicians and patients are being tested. In the Serious Illness Care Program, Lakin and colleagues found that communication skills training for primary care clinicians led to improved documentation and the comprehensiveness of goals-of-care discussions [38]. A patient activation intervention that empowers patients with heart failure to initiate goals-of-care discussions has also been found to be feasible and beneficial to both patients and clinicians [39]. Dougherty and colleagues found that patients viewed the over-the-phone coaching and the pre-visit patient activation outline to be facilitators of goals-of-care discussions. However, these kinds of interventions are inconsistently available to people with serious illness in most healthcare systems. Hence, there remains a great need to increase capacity to introduce end-of-life communication earlier for people with serious illness.

The patient- and family- related challenges of integrating end-of-life communication are not limited to the four that are identified in this study. Moreover, the identified challenges intertwine with other barriers posed by the healthcare system and present clinicians with a moral dilemma. Specifically, clinicians have a duty to share information and present services that could benefit patients and caregivers such as end-of-life discussions that have been found to improve patient and caregiver reported outcomes [40]. However, clinicians ought to respect individual preferences to wait to engage in end-of-life discussions. Our findings suggest that although patients and caregivers may seem hesitant to discuss end-of-life care, they have needs and concerns that are not being expressed. In a qualitative study of patients’ perspectives on physician behavior during end-of-life discussions, Abdul-Razzak and colleagues found that assessing a patient’s readiness to engage may be important before initiating end-of-life discussions [41]. As assessing readiness can be a challenge for physicians, they found that patients might prefer to be directly asked to have end-of-life discussions. Together, these findings stress the importance of integrating end-of-life communication as an iterative process, particularly in a highly variable illness such as heart failure, to ensure that patients and caregivers are provided with opportunities to discuss concerns and challenges at different points along the illness trajectory.

### Limitations

The findings should be interpreted in the context of several limitations. First, the clinical nature of heart failure presents unique challenges for end-of-life communication. However, all patients had multiple chronic conditions and it is uncertain to what extent this study captured how the complexity of multimorbidity affected engaging in end-of-life communication. Nonetheless, many older adults with heart failure have multiple chronic conditions, and therefore, our sample well represents the older adult population. Second, in any qualitative study it can never be determined the extent to which participants were willing to share their thoughts and insights. This is potentially of greater relevance given the sensitivity and discomfort that surrounds end-of-life discussions. However, it is also possible that participants felt comfortable confiding in a situation where their identity would remain anonymous which appeared to be the case as participants shared their concerns. It is also possible that the presence of caregivers in dyad interviews could have influenced the data, which was unaccounted for in our study. We found dyad interviews to generate rich discussions between patients and caregivers, however, this study cannot speak to how the data would differ had all interviews been solo interviews. Third, patients and family members were recruited from one outpatient heart failure clinic. This clinic also operates as a specialized heart failure clinic, which in our study setting means that patients are cared for by an interdisciplinary team of clinicians with training in heart failure management, which reflects the in-depth knowledge of heart failure management that participants had. This limits the transferability of our findings as not all patients may reflect the sample recruited in this study and the care setting. Lastly, participants in this study were predominantly Caucasian, and therefore, largely reflect the perceptions of people from one ethnic background.

## Conclusion

This study identified four challenges of engaging in end-of-life communication from the perspective of older adults with advanced heart failure and family caregivers. These findings provide useful clinical insights that can inform approaches to integrate end-of-life communication into serious illness care. The findings highlight a need for end-of-life communication to begin earlier in the course of illness to better support patients and caregivers during a period of decline as well as for communication to occur iteratively for those who may not be ready to engage. Future research is needed to explore and test alternative communication approaches that effectively unravel the challenges of illness among older adults with serious illness and their caregivers earlier in the illness trajectory.

## Supplementary information

**Additional file 1.** Sample interview questions

## Data Availability

The datasets generated and/or analyzed during the current study are not publicly available due to restrictions found in the research ethics approval. Study participants did not provide consent to have transcripts of their data shared publicly. De-identified data for researchers who meet the criteria for access to confidential data may be requested from the Mount Sinai Hospital Research Ethics Board. For more information, please contact JENNIFER.IM@MAIL.UTORONTO.CA.
