# Supplementary material for: “Whatever happens, happens” challenges of end-of-life communication from the perspective of older adults and family caregivers: a Qualitative study
Source: BMC Palliat Care. 2019 Dec 12;18:113. doi: 10.1186/s12904-019-0493-7 (PMC6909516; doi:10.1186/s12904-019-0493-7)
Supplement: Supplementary file 1 — Additional file 1. Sample interview questions [file 12904_2019_493_MOESM1_ESM.docx]

| **Supplementary File 1: Sample interview questions** |
| --- |
| 1) I understand that you’ve been diagnosed with heart failure. When were you first diagnosed? How did you find out?  Prompt: What issues have you had with your heart?  Prompt: What do you understand about your health?  Prompt: What have healthcare professionals told you about your heart? |
| 2) What has your experience with heart failure [or other health issues] been like?  Prompt: have you had to go to the emergency department or been hospitalized? What was it like? |
| 3) What are some things you enjoy doing in your day-to-day life?  Prompt: have you experienced any challenges/what are you unable to do due to your heart/health issues? |
| 4) Can you tell me about some things that are important to you in your life? |
| 5) Have you thought about the end-of-life before? In what ways? |
| 6) Have you talked about end-of-life care before?  Prompt: How did the conversation go? |
| 7) Hypothetically, if you were to decline in the next few weeks, what would you say are important to you?  Prompt: What would your preferences for the end-of-life be? |
| 8) Have you discussed end-of-life care with your healthcare provider or family members?  Prompt: Can you describe the process/conversation to me? |
| 9) Why do you think you and your provider haven’t talked about the future or the end-of-life? |
| 10) Do you think it’s important that your healthcare providers know what’s important to you? |
